# Pregnancy outcome in Refsum disease: Affected fetuses and children born to an affected mother

**DOI:** 10.1002/jmd2.12020

**Published:** 2019-03-14

**Authors:** Patricia Dubot, Léonardo Astudillo, Guy Touati, Julien Baruteau, Pierre Broué, Sandrine Roche, Frédérique Sabourdy, Thierry Levade

**Affiliations:** ^1^ Laboratoire de Biochimie Métabolique Centre de Référence en Maladies Héréditaires du Métabolisme, Institut Fédératif de Biologie, CHU de Toulouse Toulouse France; ^2^ INSERM UMR1037 CRCT (Cancer Research Center of Toulouse) Toulouse France; ^3^ Service de Médecine Interne Centre de Référence en Maladies Héréditaires du Métabolisme, CHU de Toulouse Toulouse France; ^4^ Département d'Hépato‐gastroentérologie pédiatrique, Centre de Référence en Maladies Héréditaires du Métabolisme Hôpital des Enfants, CHU de Toulouse Toulouse France

**Keywords:** peroxisome, phytanic acid, pregnancy, Refsum disease

## Abstract

We describe the case of a young woman, from a consanguineous family, affected by adult Refsum disease (ARD, OMIM#266500). ARD is a rare peroxisomal autosomal recessive disease due to deficient alpha‐oxidation of phytanic acid (PA), a branched‐chain fatty acid. The accumulation of PA in organs is thought to be responsible for disease symptoms. The patient presented only bilateral shortening of metatarsals and has been treated with a low‐PA diet. She is homoallelic for the c.135‐2A > G mutation of *PHYH*, and she married her first cousin carrying the same mutation. She was pregnant seven times and had two homozygous girls. Due to a potential exacerbation of the disease during the third trimester of pregnancy, her weight and plasma PA levels were monitored. No specific events were noticed for the mother during the pregnancies and postpartum periods. This case also raised the question of potential exposure to PA (and its subsequent toxicity) of a homozygous fetus in a homozygous mother. Despite modestly elevated plasma concentrations of PA at birth (<30 μmol/L), the two affected girls did not present any specific sign of ARD and have so far developed normally. As only a few determinations of plasma PA levels in the mother could be performed during pregnancies, showing mild elevations (<350 μmol/L), it remains difficult to conclude as to a possible transplacental crossing of PA.

## 1 INTRODUCTION

Adult Refsum disease (ARD, OMIM#266500) is a rare autosomal recessive peroxisomal disorder. The main symptoms are retinitis pigmentosa progressing to blindness, polyneuropathy, and cerebellar ataxia. Additional features include short metacarpals and metatarsals, hearing loss, anosmia, ichthyosis, cataract, cardiac arrhythmia, and elevated protein level in cerebrospinal fluid. The age of onset of the symptoms varies from early childhood to the third decade of life.^1^

This disease is characterized by the accumulation of phytanic acid (PA) in different tissues (especially the adipose tissue and nervous system) and body fluids.^2^ PA is a saturated branched‐chain fatty acid, which is specifically oxidized in the peroxisomes, first through alpha‐oxidation into pristanic acid and then by beta‐oxidation.^3^ ARD results from a deficiency of PA alpha‐oxidation.^4^ The majority of patients (>90%) have mutations in the *PHYH* gene encoding the peroxisomal enzyme phytanoyl‐CoA 2‐hydroxylase.^5^ ARD can also be caused by mutations in the *PEX7* gene encoding the PTS2 receptor.^6^

In humans, PA comes from dietary sources such as dairy products and ruminant fat. PA is transported by circulating lipoproteins^7^ and is mostly stored in the adipose tissue. Treatment of ARD patients is based on PA‐restricted diet and/or physical extraction by plasmapheresis or apheresis. A study of 13 patients treated with long‐term dietary therapy showed a decrease of 89% of circulating PA levels and an improvement of clinical signs, highlighting the efficiency of this therapy.^8^

When plasma levels of PA are high (>1000 μmol/L), acute presentations of ARD can occur with an exacerbation of symptoms such as sudden visual deterioration. These manifestations are usually caused by rapid weight loss, poor dietary compliance, surgical or medical admission, or infections.^8^ Pregnancy also represents a stressful period, especially during the third trimester, when lipolysis can occur due to insulin resistance.^9^ This may result in an acute mobilization of PA^10^ and an exacerbation of symptoms. Pregnancy outcomes in a homozygous female have already been described; her heterozygous fetus developed normally.^11^

Here, we describe the first published case of a homozygous woman affected by ARD, who was repeatedly pregnant with homozygous fetuses.

## 2 CASE HISTORY

**Figure 1:**
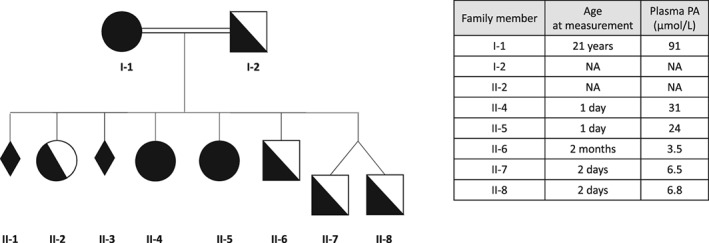
Patient's family pedigree. Plasma PA levels in the various family members are also indicated. NA, not available

A young woman, born to a multiconsanguineous family with several members suffering from ARD, was diagnosed with ARD at the age of 21. The first clinical examination revealed the characteristic bone abnormalities in both feet (shortening of the metatarsals). No neurological sign was observed. Diagnosis was established both biochemically (plasma PA 91 μmol/L; controls <6.5 μmol/L; no elevation of pristanic acid or very long chain fatty acids) and molecularly. She is homoallelic for the familial mutation of *PHYH* gene c.135‐2A > G. She was then treated with dietary restriction of PA. Almost concomitantly, she became pregnant for the first time. She married her first cousin, who is heterozygous for the same *PHYH* mutation (Figure 1). Due to a high risk of a homozygous ARD fetus, a prenatal diagnosis was carried out, which revealed a homozygous status; this was followed by a miscarriage a few days after trophoblast biopsy.

Prenatal diagnosis for the second pregnancy revealed a heterozygous status and the pregnancy proceeded without any special events. For the third pregnancy, a homozygous fetus was prenatally diagnosed, which led to a medical termination of pregnancy. For the fourth pregnancy, in spite of a prenatally diagnosed homozygous fetus, the parents wanted to continue the pregnancy. For the subsequent three pregnancies, no prenatal diagnosis was performed and one additional homozygous girl was born. As a result, two children are affected by ARD (patients II.4 and II.5, Figure 1).

**Figure 2:**
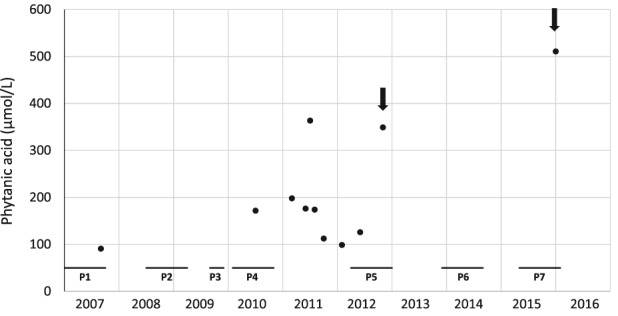
Plasma PA levels in the female patient (I‐1). All pregnancies (P) are numbered (horizontal bars). Arrows indicate the highest values recorded during pregnancies

During all pregnancies, dietary therapy of the mother was essentially unchanged (although no detailed dietary history was recorded), with monitoring of body weight and plasma PA levels. Only few measures of plasma PA were obtained (Figure 2) because of the lack of compliance of the patient. Two values were higher than usual during the last trimester of the fifth and seventh pregnancies, reaching 349 and 511 μmol/L (Figure 2, arrows); no specific clinical event was noticed during these periods. Also, despite some excessive weight gain (+10 kg during the first 4‐5 months of the fourth pregnancy), the mother presented no adverse event during delivery and postpartum periods.

As for the fetal development management of homozygous fetuses, ultrasound examinations did not reveal abnormalities. Fetal viability, biometric indices, morphological parameters, and annexes were all normal. The two ARD‐affected girls were born without special events (in particular, no shortened metatarsals). Their plasmatic PA levels at birth were slightly elevated (Figure 1). The newborns were not breastfed but fed with poor PA‐containing milk and were immediately treated with a low‐PA diet.

Clinical and biological evaluations were regularly carried out for girl II.4 during the first 2 years. Whereas the PA level returned to normal values at 3 weeks of age, it slightly increased at age 9 months, reaching 158 μmol/L at age 5 years. Clinically, only a nystagmus was observed that spontaneously regressed. Regarding girl II.5, her plasma PA level was normal at 4 months of age but increased up to 219 μmol/L at 3 years, possibly because of diet deviation. At last evaluations, patients II‐4, aged 5 years, and II‐5, aged 3 years, had normal physical and psychomotor development.

## 3 DISCUSSION

This seems to be the first documentation of affected ARD children born to a homozygous mother. The affected mother belongs to a consanguineous family that carries the c.135‐2A > G mutation and the homozygous polymorphism c.153C > T in exon 3. The mutation in the splice acceptor site of intron 2 results in skipping of exon 3 (c.135‐245del), leading to a detectable but smaller protein that is much less expressed than the wild‐type protein likely due to premature degradation. Moreover, this truncated protein has been reported to lack enzymatic activity.^5^ The plasma levels of PA at diagnosis in the present case (I.1) were not as high as those previously observed in a female patient carrying the same mutation and the same polymorphism, and who presented a late‐onset form of the disease (at the age of 32 years) with rapidly progressive blindness.^12^ The patient herein described seems to naturally have a low‐PA diet, which could explain her PA plasma levels. Whether the c.153C > T polymorphism impacts the clinical manifestations remains unknown. Phenotypic variation among patients with identical genotype is already a known feature of ARD^13^.

This observation raises two main questions: the management of an affected ARD woman during pregnancy and the possible teratogenic action of PA. The pregnancy management of an ARD female patient with a heterozygous fetus has already been reported.^11^ In this situation, the risk for the fetus remains low because of the residual phytanoyl‐CoA hydroxylase activity. However, for the mother, the risk of exacerbation of ARD is higher, on account of changes in lipid metabolism during pregnancy, especially lipolysis in the third trimester. Thus, it seems that the low‐PA diet and measures to avoid lipolysis are efficient for the pregnant patient. In the presently described homozygous patient, management of the pregnancies relied on a dietary restriction with clinical and biological monitoring. Despite the rather modest elevation of PA (Figure 2, arrows), no clinical event was noticed.

The present family case report may also highlight the issue of a possible transplacental crossing of PA and of its potential toxicity. Indeed, the accumulation of PA is believed to be the cause of the clinical symptoms in ARD. Should PA be transported across the placenta, the question arises as to whether a homozygous ARD fetus (ie, with deficient alpha‐oxidation) would be at risk of developing early abnormalities because of in utero exposure to pathologically high concentrations of PA (due to ARD in the mother). One could postulate that, as a fatty acid, PA is able to cross the placenta; however, the great difference between fetal calf and adult bovine PA serum levels may indicate that PA does not cross the placental barrier (at least in cows).^14^^,^ 15 Here, due to the patient's lack of compliance, only a few measures of PA were obtained, making the assessment of potential fetal exposure difficult. Nevertheless, for both homozygous affected siblings, the plasma PA level at birth was above normal, but rapidly decreased thereafter. These findings suggest some extent of a transplacental crossing of PA, followed by a normalization of the circulating levels after birth due to the absence of exogenous supply. The homozygous children were born without any defects or ARD manifestations; in addition, none of them have developed ARD symptoms so far. As they are only 5 and 3 years old, further follow‐up is needed to exclude late‐onset consequences or minor neurodevelopmental abnormalities. We cannot rule out the possibility that the absence of any symptoms in the affected siblings is explained by the fact that PA levels in their mother were not exceedingly high (with two exceptions, <200 μmol/L).

Is PA teratogenic? To our knowledge, no experimental studies directly addressing the question of a possible PA transplacental crossing and fetal toxicity in humans have been published. The murine model harboring a disruption of the phytanoyl‐CoA 2‐hydroxylase gene (*Phyh* ^*−/−*^ *)* displays no abnormalities. These mutant mice are reported to be fertile and their offspring viable.^16^ In rodents, PA is believed to act as a natural agonist for the retinoid RXR receptor at physiological concentrations.^17^, ^18^ Coadministration of PA and ligands of retinoid receptors to gestating mice has been reported to increase teratogenesis.^19^ In humans, besides the possible but still questionable role of PA in prostate cancer,^20^ the fetal toxicity of PA remains unknown. Only in one instance, fetal exposure to PA has been postulated for a young boy affected by a very early‐onset ARD hypothetically born to a homozygous mother.^21^

In conclusion, as previously highlighted,^11^ this case of a homozygous woman suffering from ARD and pregnant with affected fetuses suggests that the risk of developing disease manifestations is higher for the mother during pregnancy than for her fetus. Whereas additional cases need to be investigated, prenatal diagnosis may not be indicated in an affected mother who follows regular dietary therapy during pregnancy and whose plasma PA levels are monitored.

## CONFLICT OF INTERESTS

Patricia Dubot has accepted reimbursement for attending symposia from Sanofi‐Genzyme, Shire, Biomarin, or Orphan companies. Frédérique Sabourdy has accepted reimbursement for attending symposia from Sanofi‐Genzyme, Shire, Biomarin, or Orphan companies. Thierry Levade has accepted reimbursement for attending symposia from Sanofi‐Genzyme, Shire, Biomarin, or Orphan companies. Sandrine Roche has accepted reimbursement for attending symposia from Sanofi‐Genzyme, Shire, Biomarin, Nestlé, Biocodex, or Orphan companies. Guy Touati, Pierre Broué, Léonardo Astudillo, and Julien Baruteau declare that they have no conflict of interest.

## AUTHOR CONTRIBUTIONS

Patricia Dubot, Léonardo Astudillo, Guy Touati, Frédérique Sabourdy, and Thierry Levade performed the literature search and analyzed the data. Patricia Dubot, Léonardo Astudillo, and Thierry Levade wrote the first draft of the manuscript. All authors were involved in revisions and approved the final version of the article. Guarantor: Thierry Levade.

## INFORMED CONSENT

Informed consent for DNA analysis was obtained from the patients.
